# The role of pregnane X receptor (PXR) in substance metabolism

**DOI:** 10.3389/fendo.2022.959902

**Published:** 2022-08-16

**Authors:** Ye Lv, Yi-Yang Luo, Hui-Wen Ren, Cheng-Jie Li, Zhi-Xin Xiang, Zhi-Lin Luan

**Affiliations:** ^1^ Advanced Institute for Medical Sciences, Dalian Medical University, Dalian, China; ^2^ Dalian Key Laboratory for Nuclear Receptors in Major Metabolic Diseases, Dalian Medical University, Dalian, China

**Keywords:** pregnane X receptor (PXR), nuclear receptor, metabolism, glycometabolism, lipid metabolism, bile acid, vitamin, endocrine homeostasis

## Abstract

As a member of the nuclear receptor (NR) superfamily, pregnane X receptor (PXR; NR1I2) is a ligand-activated transcription factor that plays a crucial role in the metabolism of xenobiotics and endobiotics in mammals. The tissue distribution of PXR is parallel to its function with high expression in the liver and small intestine and moderate expression in the kidney, stomach, skin, and blood-brain barrier, which are organs and tissues in frequent contact with xenobiotics. PXR was first recognized as an exogenous substance receptor regulating metabolizing enzymes and transporters and functioning in detoxification and drug metabolism in the liver. However, further research revealed that PXR acts as an equally important endogenous substance receptor in the metabolism and homeostasis of endogenous substances. In this review, we summarized the functions of PXR in metabolism of different substances such as glucose, lipid, bile acid, vitamin, minerals, and endocrines, and also included insights of the application of PXR ligands (drugs) in specific diseases.

## Introduction

**Figure 1:**
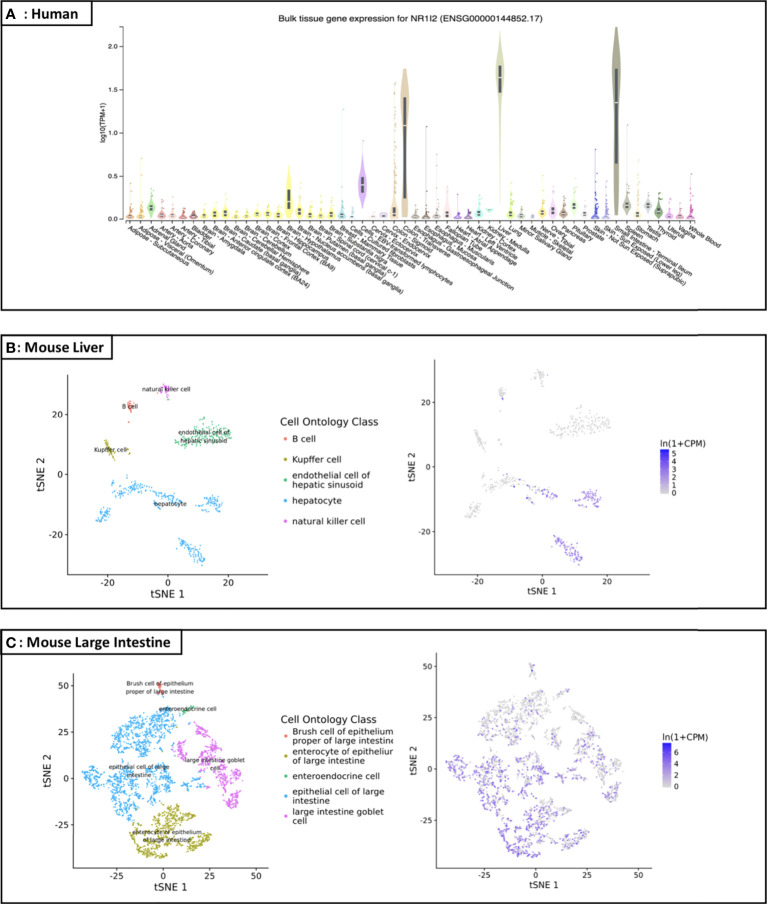
Distribution map of PXR in human and specific organs of mice **(A)** Expression profile of the *NR1I2* (*PXR*) gene in human: According to the GTEx database (https://gtexportal.org/home/gene/NR1I2), the *NR1I2* (*PXR*) gene is highly and specifically expressed in small intestine, liver, rectum, and colon, while its expression in other organs/tissues is either low or undetectable. TPM on the vertical axis represents the transcript quantification value, and the horizontal axis represents different tissues (TPM: transcripts per kilobase of exon model per Million mapped reads; tSNE: t-distributed stochastic neighbor embedding); **(B)** Liver cell scRNA-seq analysis demonstrating that mouse PXR mRNA is highly expressed in the liver, especially in hepatocyte; **(C)** Large intestine cell scRNA-seq analysis demonstrating that mouse PXR mRNA is expressed in the large intestine, especially in epithelial cell and enterocyte of epithelium.

As a member of the nuclear receptor (NR) superfamily, pregnane X receptor (PXR; NR1I2) is a ligand-activated transcription factor first reported in 1998 and named based on its activation by endogenous pregnane 21-carbon steroids (1, 2). PXR is highly distributed in small intestine, liver, rectum, colon and bladder, while its expression in other organs and tissues is either moderate, low or undetectable (3), and the statistics from the GTEx and Tabula Muris databases also support this view (**Figure 1**) (4, 5). PXR can be activated by numerous chemical compounds. Besides pregnane, steroid hormones, bile acids and other endobiotic chemicals, various clinical drugs (e.g., statins, antidepressants, anticonvulsants) and environmental pollutants have been demonstrated as PXR ligands (**Table 1**) (35–39). Activated PXR, through direct binding to the genomic regions or indirect crosstalk with other transcriptional factors, controls various genes involved in biotransformation, transport, inflammation, oxidative stress and etc. (35).

**Table 1:** The agonists of PXR ligand.

		Agonist	Cell line/Species
**Bile acids**	Bile acids **(**Cross-species)	12-Ketolithocholic acid; 7-Ketolithocholic acid; 7-Ketodeoxycholic acid; 7,12-Dietolithocholic acid; Cholic acid; Hyodeoxycholic acid; Lithocholicacid; Glycocholic acid; Lithocholic acid-3-sulfate; Glycolithocholic acid; Taurochenodeoxycholic acid; Taurohyodeoxycholicacid; Lithocholic acid acetate; Lithocholicacid acetatemethylester (6)	Cross-species
**Hormones**	Steroids/Steroid hormones	Pregnenolone, Progesterone, Estradiol, Mifepristone, Cyproteroneacetate, Spironolactone, 5β-pregnane-3,20-dione, IncisteroloA5 and A6 (7–9)	HepG2 cell line
	Glucocorticoid	Corticosterone (7); Dexamethasone (10)	HepG2 cell line
**Clinical Drugs**	Antifungal agents	Clotrimazole (11)	Zebrafish
	Antibiotic	Sulfadimidine (8); Triacetyloleandomycin (8); Rifampin; Rifaximin (12)	HepG2 cell line
	Drugs for primary biliary cirrhosis	Budesonide (13)	Human
	Lipid-lowering drug	SR12813; Atorvastatin; Mevastatin (14)	Human
	HMG-CoA reductase inhibitors	Rosuvastatin (15)	HepG2 cell line
	Drugs for coronary heart disease	Tan IIA (16)	HepG2 cell line
	Antitumor drugs	Paclitaxel/Taxol (17)	LS174T cell line
	Antidepressants	Hyperforin (18)	HepG2 cell line
	Anticonvulsants	Phenobarbital; Pheytoin; Carbemazepine (18)	HepG2 cell line
	Antiarthritics	Sulfinpyrazone (8)	HepG2 cell line
	Antihistamines for motion sickness	Meclizine (19)	Human
	Metabolites of the antimalarial drug mefloquine	Carboxymethyl fluoroquine (20)	LS174T and HepG2 cell line
	Thiazolidinediones	Troglitazone (7)	HepG2 cell line
	Anti-HIV drugs	Ritonavir; Efavirenz (21)	hPXR mice
**Others**	Environmental Factors	Nonylphenol (22); Tributyl citrate (23); Bisphenol A (24)	hPXR mice; LS174T cell line
	Synthetic (pesticides, chemical products)	Chlordane (7); Patchouli alcohol (25); Prochloraz (26); Aroclor 1260 (27)	HepG2 cell line; hPXR mice; Human
	Fragrances	Piperine (28, 29); Anisomycin (30)	hPXR mice; HepG2 cell line
	PXR and CAR dual agonists	CITCO (31)	hPXR mice; HepG2 cell line
	Cyclohexene-type amides	Nigramide C (32)	hPXR mice
	Lead compounds	Tangshenoside II (16, 33)	HepG2 cell line
	PXR weak agonists	Resveratrol (34)	mPXR Mice

PXR is unique among NRs on account of its broad ligand spectrum, which make it a perfect tool for sensing changes in the external chemical environment. Although originally identified as a receptor for exogenous substances, PXR is now recognized as an equally important receptor for endogenous substances and plays a variety of functions in the metabolism of these substances. Many studies have shown that PXR is involved in a range of physiological and pathological processes through regulating metabolism of a large group of substances. In this review, we summarized the functions of PXR in substance metabolism in aspects of glucose and lipid metabolism, bile acid circulation, and endocrine homeostasis, and also included insights of the application of PXR ligands (drugs) in specific diseases.

## The transcriptional regulatory characteristics of PXR

**Figure 2:**
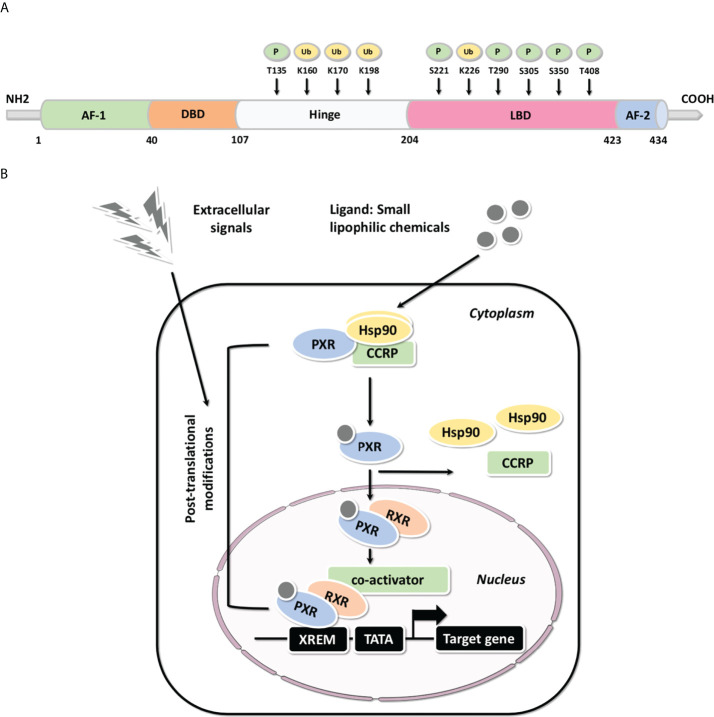
The structure and molecular mechanisms associated with PXR **(A)** Common structure of metabolic nuclear receptor and the post-translational modifications of PXR protein structure. The domain structure of metabolic nuclear receptor is presented, including the typical N-terminal non-ligand-dependent AF-1, a highly conserved DBD, a less conserved hinge region, a C-terminal LBD and AF-2; PXR may be modified by phosphorylation and ubiquitination through protein-protein interactions, thus, reported phosphorylation and ubiquitination are highlighted (P: Phosphorylation; Ub: Ubiquitination). **(B)** The molecular mechanisms of PXR-mediated gene activation: Molecular analysis based on both *in vivo* and *in vitro* models have systematically illustrated the mechanism of PXR activation.

**Figure 3:**
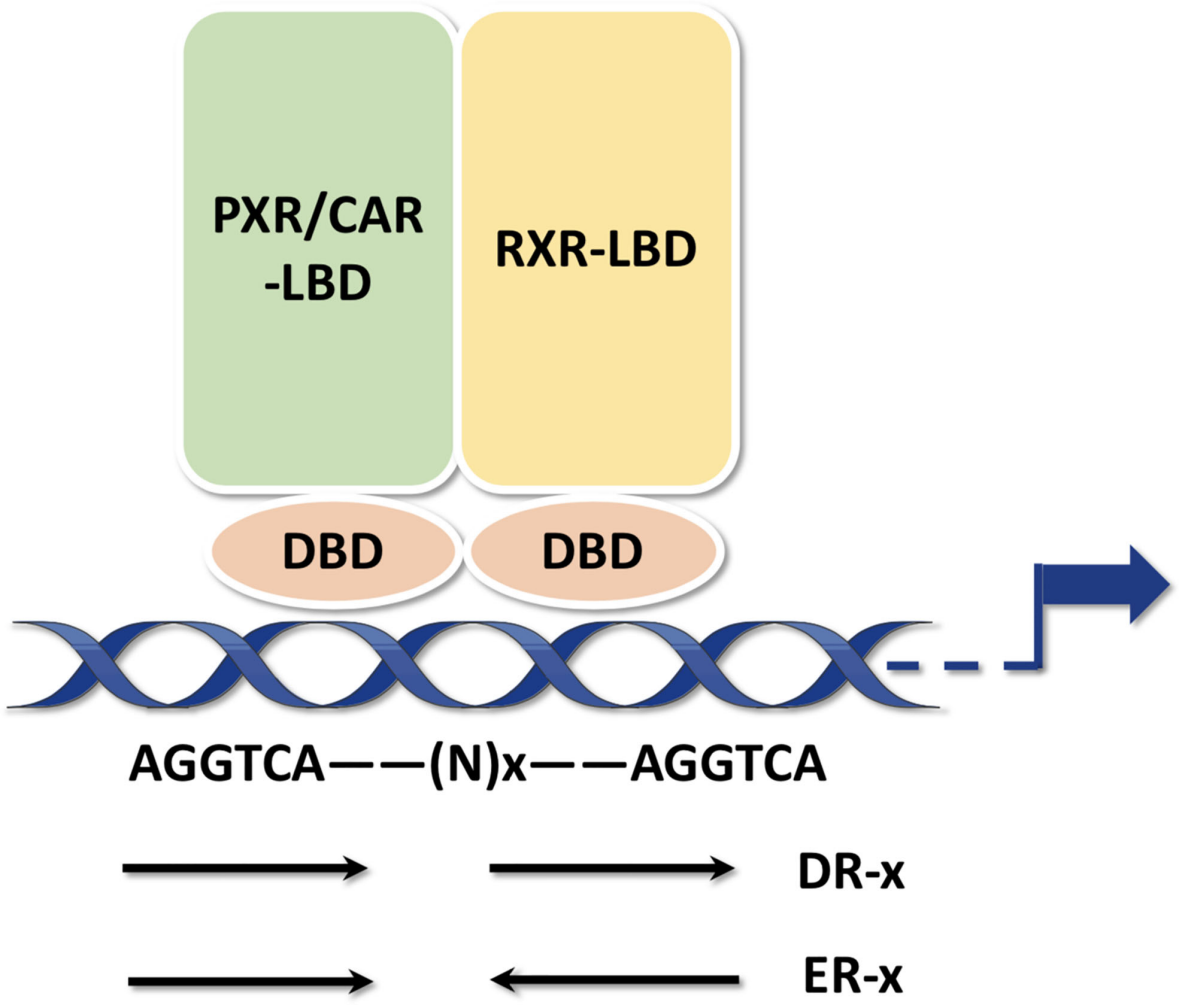
PXR response elements PXR binds as heterodimers with RXR to repeats of the nucleotide hexamer AGGTCA with variable spacing. The hexamers can be arranged either as DRs or ERs.

PXR share a common protein structure with most NRs which consists of a typical N-terminal non-ligand-dependent activation function 1 (AF-1), a highly conserved DNA-binding domain (DBD), a less conserved hinge region, a C-terminal ligand-binding domain (LBD) and an activation function 2 (AF-2) (**Figure 2A**) (2, 3, 40). It has been reported that PXR can be modified by acetylation, phosphorylation, ubiquitination, and SUMOylation through protein-protein interactions (**Figure 2A** **),** indicating that PXR is implicated in posttranslational modifications which may ultimately affect its transcriptional regulation and metabolic detoxification process. The interaction centered by PXR will illustrated the multifunctional property of it in different signaling pathways (41). Being part of a chaperone protein complex consisting of heat shock protein 90 (Hsp90) and CAR cytoplasmic retention protein (CCRP), PXR is predominantly localized in the cytoplasm (42). After activation by ligand binding, PXR is transferred from the cytoplasm to the nucleus and forms a heterodimer with retinoid X receptor (RXR). All in all, molecular analysis based on both *in vivo* and *in vitro* models have systematically revealed the mechanism of PXR activation (**Figure 2B**) (43, 44). After recruiting a large number of co-activators, the DBD domain of PXR promotes the DNA binding specificity of PXR through two highly conserved zinc finger motifs as well as the P- and D-box motifs. PXR binds as heterodimers with RXR to repeats of the nucleotide hexamer AGG/TTCA with variable spacing (45) (**Figure 3**). PXR functions as a trans-factor and regulates its downstream target genes by binding to specific promoter DNA reaction elements. Initial studies suggested that the PXR/RXR co-activation complex binds only to direct repeat sequences in the enhancer regions of target genes, such as DR3 (directed repeat 3) (46).

However, Drocourt et al. found that PXR/RXR heterodimer not only binds DR3 i found that PXR/RXR heterodimer not only binds DR3 in the enhancer region of the human CYP3A4 gene but also acts on the ER6 (everted repeat 6) element. The PXR-bound DR3 and ER6 are highly conserved and generally contain AG(G/T)TCA or TGA(A/C)CT sequences (47, 48). By binding to DR3 and ER6, activated PXR/RXR heterodimer promotes transcriptional regulation of many genes in the cytochrome P450 3A (CYP3A) family, the most abundant, clinically significant group of cytochrome P-450 isoenzymes, such as CYP3A1, CYP3A2, CYP3A23, CYP3A4, CYP3A6, CYP3A7. CYP3A4 is a major target gene for PXR and involved in 60% of drug transport *in vivo*. Although, mouse genes (e.g. Cyp3a23, Cyp3a1) are absence in humans, but they are considered as clinically significant. Recent studies have revealed that PXR can bind not only to DR3 and ER6 in the promoter region of its target genes, but also to other response elements. Geick et al. reported that PXR/RXR heterodimers can bind to three types of DR4, with DR4(I) and DR4(III) having the highest affinity. The binding of PXR to DR4 is essential for transcription of certain downstream target genes, such as the multi-drug resistance gene 1 (MDR1) and CYP2B3 families (49). Jeske et al. found that PXR can directly bind DR4 and ER8 on the first intron at the 5’ end of sphingomyelin phosphodiesterase acid-like (SMPDL) 3A, and in the presence of non-ligands can then bind (50). As a newly discovered hepatic phospholipase, SMPDL can activate the carbamate precursor drug CS-917 and serves as a promising candidate for the treatment of type 2 diabetes (51). It has also been found that PXR/RXR can bind to ER8 in the promoter region of multidrug resistance protein 2 (MRP2) and promote MRP2 protein transcriptional expression. In summary, the binding elements of PXR/RXR on DNA are divided into: direct repeats (DR4, DR5, DR9, DR9, DR14, DR19), everted repeats (ER6 and ER8), and inverted repeats (IRs) (52).

**Figure 4:**
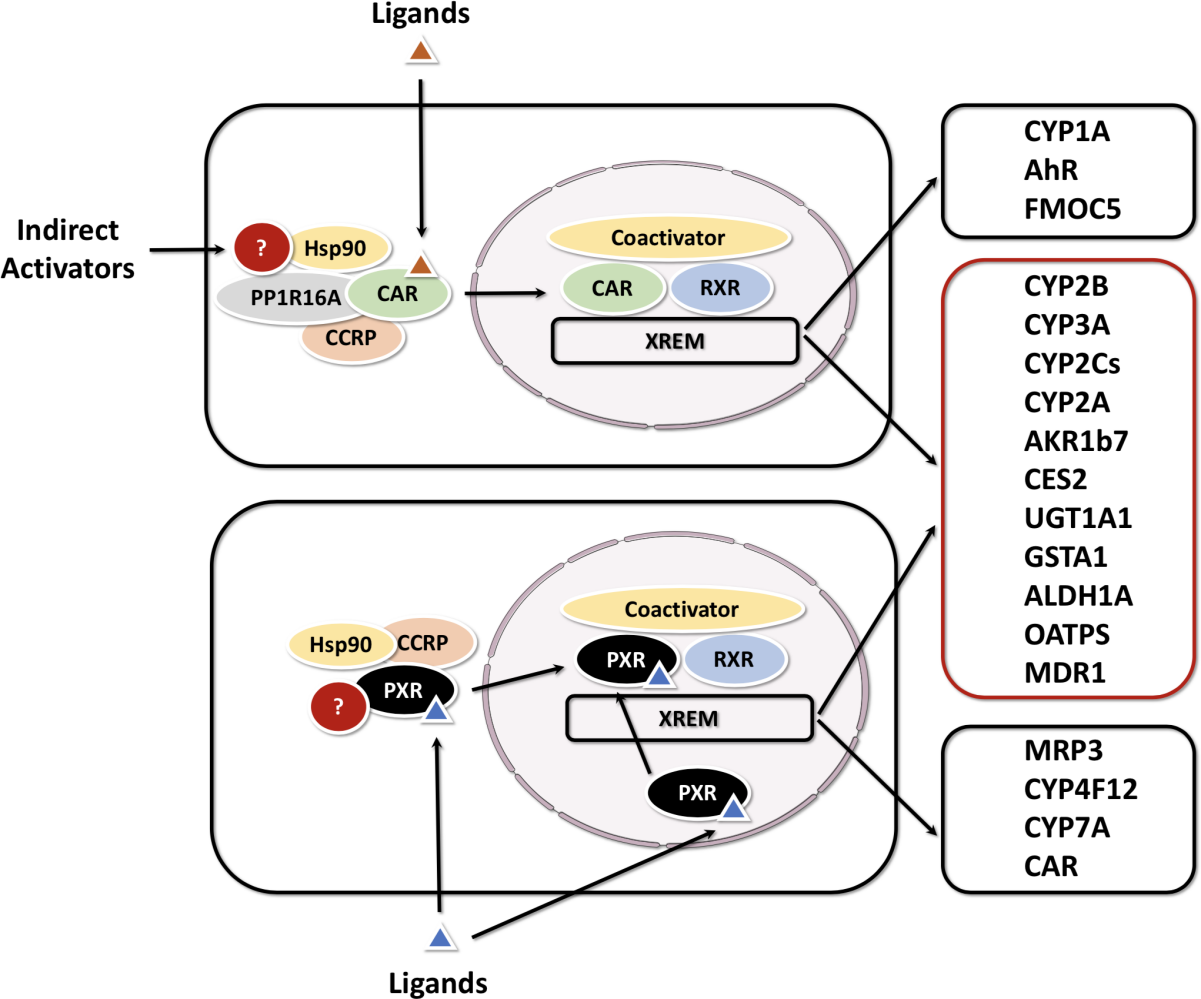
Activation mechanisms and target genes of CAR and PXR The activation of PXR is purely ligand dependent, while CAR can be activated by either direct (ligand binding) or indirect mechanisms. CAR and PXR shared target genes are grouped in a red box, CAR or PXR-specific targets in a black box.

In addition to xenobiotic receptors above, PXR and CAR can also collaboratively exhibit promiscuous xenobiotic activation. They govern the transcription of a broad spectrum of distinct and overlapping genes encoding phase I, phase II drug-metabolizing enzymes (DMEs), as well as uptake and efflux transporters (53–55). Notably, CAR and PXR share significant cross-talk in both target gene recognition by binding to the similar xenobiotic responsive elements in their target gene promoters, and in accommodating a diverse array of xenobiotic activators (56, 57). Coordinately, CAR and PXR regulate a largely overlapping set of xenobiotic metabolizing genes. These target genes include several CYPs (i.e. CYP3A4, CYP2B6, CYP2Cs, and CYP2A6) (58, 59), UGTs (i.e. UGT1A1, UGT1A6, and UGT1A9) (60, 61), GSTs, and SULTs; as well as drug transporters such as MRPs, MDR1 and OATPs (62). On the other hand, CAR displays unique activation mechanisms compared with PXR and other orphan receptors, involving both direct ligand binding and indirect ligand-independent pathways (**Figure 4**) (63).

The development of structural genomics has provided insight into the structural basis of NR-regulated transcription. Watkins et al. completed the first X-ray crystallographic analysis of PXR LBD (64). Similar to the LBDs of other members in the NR superfamily, the PXR LBD contains a triple helix sandwich (H1/H3, H4/H5/H8, H7/H10) (65, 66). A fragment containing 45 amino acids is inserted between helix 1 (H1) and helix 3 (H3) as a β-sheet in the PXR LBD and forms one of the five chains of β-sheet in the PXR structure. However, other NRs contain only two or three β-sheet chains. Part of helix 2 (H2) in the PXR LBD is replaced by a β-fold, forming a spherical ligand-binding cavity with H1 and H3, also known as the “ligand-binding pocket”. With the potential for flexible expansion and contraction, a ring-like structure in the PXR LBD is formed by β2, β3 and β4-expanded helix 6 (H6) which graphically serves as a “ligand-binding pocket”. The “ligand binding pocket” has a uniform distribution of hydrophobic amino acid residues on its surface, allowing the ligand to maintain equilibrium in any orientation through hydrogen bonding and van der Waals forces (67). The above molecular features allow PXR to recognize a wide range of xenobiotics.

Although PXR can accept a wide variety of ligands, the degree of ligand binding is species-specific. For example, pregnenolone-16α-carbonitrile (PCN) activates the rodent PXR but not the human PXR, and SR12813 and rifampicin activate human PXR at high levels but not rodent PXR. In organisms, xenobiotic promotion of PXR activation causes more extensive metabolic changes involving downstream target gene transcription other than the direct involvement of activated PXR in xenobiotic metabolic processes. In addition, PXR activation has also been reported associated with a variety of diseases, therefore, clinical application of PXR ligands requires consideration not only of individual patient differences, but also of changes in drug efficacy in the body when administering different drugs to patients.

## PXR in glucose metabolism

Increasing evidence indicates that PXR activation functions in glucose homeostasis. Blood glucose concentration maintains relatively constant by hormones (insulin, glucagon, glucocorticoids etc.) that regulate the activity of key enzymes involved in various pathways of glucose metabolism. In mouse primary hepatocytes, human hepatoma HepG2 and Huh7 cells, PXR activation inhibits the expression of glucose-6-phosphatase (G6Pase) and phosphoenolpyruvate carboxykinase (PEPCK), two key enzymes inhibiting gluconeogenesis (68–70). Kodama et al. showed that PXR regulates gluconeogenesis by interacting with forkhead box protein O1 (FOXO1), cAMP-response element binding protein (CREB) and hepatic nuclear factor 4 (HNF4) (70). HNF4, together with the nuclear receptor co-activator PGC-1α, positively regulates gluconeogenesis. Bhalla *et al.* found that PXR competes with HNF4 for PGC-1α, thereby inhibiting gluconeogenesis (68). *In vivo* experiments also confirmed the plausibility of these results. FOXO1 functions as a G6Pase and PEPCK activator in insulin deficiency. Under normal conditions, after binding to the insulin response sequence (IRS), insulin repatriates FOXO1 from the nucleus *via* PI3K-Akt pathway, thereby inhibiting G6Pase and PEPCK expression and FOXO1-mediated transactivation of gluconeogenesis. Glucagon increases intracellular cAMP formation, which activates protein kinase A (PKA), which in turn activates CREB that binds and regulates G6Pase and PEPCK transcription. On the other hand, PXR inhibits CREB binding to homologous binding elements, thus preventing the transcription of glucagon-activated G6Pase and thereby inhibiting the gluconeogenic process (69). Nakamura et al. demonstrated that Pxr activation by PCN inhibited hepatic G6pase and Pepck expression in rats and mice (71). Similarly, the expression of G6pase and Pepck in the liver of VP-hPXR mice treated with rifampicin was also decreased (72). But these did not occur in Pxr-knockout mice. The downregulation of G6pase and Pepck expression may indicate that Pxr activation reduces hepatocyte glucose output, potentially improving glucose homeostasis in type 2 diabetes. Studies have also shown inconsistent gluconeogenic responses in the liver following Pxr activation (15, 73). As mentioned earlier, G6pase and Pepck expression was inhibited in mouse liver and human hepatoma cells following Pxr activation. However, rifampicin treatment of human primary hepatocytes for 6 h was able to induce the 2 times expression of G6Pase; whereas rifampicin treatment of human primary hepatocytes for 24 h resulted in a 30% reduction in G6Pase mRNA compared to the control group. Another study showed that simvastatin treatment of human hepatocytes for 24 h resulted in 7 times increase in PEPCK1 mRNA expression compared to the control group. Gotoh and Negishi et al. found that the PXR in human hepatocyte can bind directly to the promoters of G6Pase and PEPCK to regulate blood glucose. There are two distinct binding sites, one is the classical direct PXR binding site, and the other is the IRS site *via* protein-protein interactions. The activation of PXR binding to the promoter requires the involvement of serum/glucocorticoid regulated kinase 2 (SGK2) dephosphorylation co-activating transcription factors. Interestingly, PXR not only alters the phosphorylation status of SGK2, but also binds to the activated SGK2 gene promoter to induce SGK2 expression (15). The mechanism of PXR-mediated regulation of human hepatic gluconeogenesis still needs further investigation.

In addition to regulating gluconeogenesis, PXR activation is also involved in the oxidative absorption of glucose. The hepatic level of glucose transporter 2 (GLUT2) mRNA was downregulated by PCN. In rat and mouse hepatocytes, PCN-mediated activation of PXR downregulated the expression of glucose transporter 2 (GLUT2) and glucokinase (GCK) indicating a detrimental role of PXR activation on glucose tolerance (74). GCK drives the phosphorylation of glucose to glucose-6-phosphate, which is the first step in glycolysis. Mutations leading to reduced GCK activity have been reported as the cause of early-onset type 2 diabetes, and GCK activators are being investigated as potential agents for type 2 diabetes (75). As a major component of green tea (–),-Epigallocatechin-3-gallate (EGCG) activated PXR and constitutive androstane receptor (CAR), accompanied by up-regulating expressions of PXR/CAR-mediated phase _2_etabolism enzymes (SULT1A1, UGT1A1 and SULT2B1b) in small intestine and liver (76). Thereby, this process can inhibit the starch digestion and improving glucose homeostasis. Therefore, EGCG has been considered as a promising PXR/CAR activator and therapeutic intervention in diabetes.

**Figure 5:**
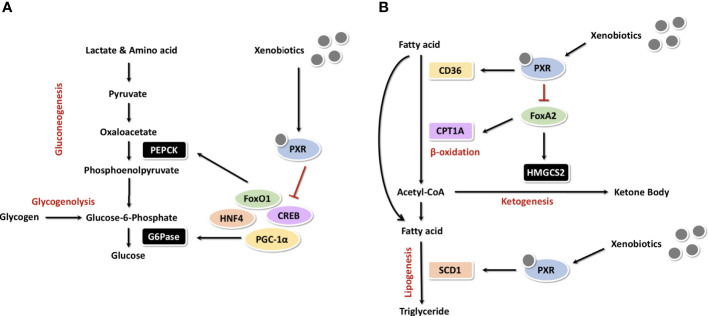
PXR in the regulation of hepatic glucose and cholesterol metabolism **(A)** As a xenobiotic sensing regulator, PXR plays a crucial role in hepatic glucose metabolism. **(B)** PXR in the regulation of hepatic cholesterol metabolism.

As for some oral hypoglycemic drugs, Shashi *et al.* showed that some oral antidiabetic agents, such as rosiglitazone and pioglitazone (thiazolidinediones, TZDs), can also activate PXR and upregulate its downstream expression of CYP3A4, UGT1A1, MDR1 and thereby may inflict undesirable consequences (77). As GLUT2 and GCK have important functions in postprandial glucose uptake, their abnormal regulation may be involved in PXR-induced postprandial hyperglycemia. Indeed, hepatic GLUT2 and GCK knockout rats developed mild hyperglycemia under normal feeding (78, 79). The regulation of GLUT2 and GCK by PXR was demonstrated in the HepG2 model. Atorvastatin reduced protein levels of GLUT2 and GCK and decreased glucose consumption and uptake in HepG2 cells. However, pravastatin had no effect on GLUT2 and GCK expression and no effect on glucose utilization. Based on *in vitro* studies, atorvastatin, simvastatin, lovastatin and fluvastatin are PXR activators, whereas pravastatin and rosuvastatin are not agonists of the PXR. At the same time, PXR knockdown or overexpression can up and down-regulate GLUT2 and GCK expression accordingly (80). Fatemeh et al. also verified these results in Pxr wild-type and Pxr knockout mice treated with PCN, where only in the wild type was the level of Glut2 protein down-regulated and glucose tolerance impaired after PXR activation (81). In a word, as a xenobiotic sensing regulator, PXR plays a crucial role in hepatic glucose metabolism (**Figure 5A**). These results indicate that the activation of PXR impairs glucose tolerance and thus PXR represents a novel diabetogenic pathway.

## PXR in lipid metabolism

Triglycerides and fatty acids are important metabolic fuels. Lipid homeostasis balances lipid uptake and synthesis with lipid metabolism and secretion. When glucose and fatty acids exceed the body’s energy requirements, they are converted to triglycerides and stored in the liver. The expression of PXR and fatty acid binding protein 4 (FABP4) were increased by Valproate (valproic acid, VPA), a widely used drug in the therapy of epilepsy, in a dose-dependent manner. On the contrary, knockdown of PXR not only reduced lipid accumulation but also impaired the induction of FABP4 by VPA. While overexpression of PXR enhanced both lipid accumulation and FABP4 expression. These results suggest that PXR-mediated expression of FABP4 is responsible for lipid accumulation caused by VPA (82).

During fasting or exercise, fatty acid β-oxidation and ketogenesis increase in adipocytes, thereby promoting ketone body synthesis and energy production. The sterol regulatory element-binding protein-1c (SREBP-1c) is a major regulator of lipogenesis. Some NRs, such as liver X receptor (LXR), HNF4 and LRH-1, control lipid homeostasis by regulating the transcriptional activity of SREBP (83–85). VP-PXR transgenic mice develop intrahepatic triglyceride accumulation and are associated with upregulation of the fatty acid translocase CD36 and certain other lipogenic coenzymes, including SCD-1 and long-chain free fatty acid elongase. CD36 is a scavenger receptor with broad ligand specificity. The activation of CD36 promotes uptake of free fatty acids from the circulatory system and may be involved in hepatic steatosis (86). The correlation between CD36 levels and the storage and secretion of triglyceride suggests that CD36 plays an initiating role in hepatic steatosis. Moreover, PXR plays an essential role in CD36 transcription. Studies have shown that CD36 is a direct target gene of PXR transcriptional regulation (72). The expression of CD36 can also be positively regulated by LXR and peroxisome proliferator-activated receptor γ (PPARγ). Therefore, CD36 should be a common transcriptional target gene of LXR, PXR and PPARγ in the regulation of lipid homeostasis (87). Studies in the cardiovascular field have shown that higher Bisphenol A (BPA) exposure has been associated with an increased risk of atherosclerosis and cardiovascular disease (CVD). hPXR but ApoE knockout model mice were used by Sui et al. to study the teratogenic effects of BPA. It indicates that PXR epigenetically regulated CD36 expression by increasing H3K4me3 levels and decreasing H3K27me3 levels in the CD36 promoter in response to perinatal BPA exposure (88).

It has also been added that PXR can be activated by efavirenz, a drug commonly used in the treatment of HIV infection and proved as PXR-selective agonist. After efavirenz-mediated Pxr activation in mice, cholesterol biosynthetic cyclooxygenase (SQLE) can be regulated as a direct transcriptional target of Pxr in addition to CD36, leading to increased lipid uptake and cholesterol biosynthesis in hepatocytes. Considering that activation of PXR signaling may induce hypercholesterolemia and cirrhosis, the combination of this finding also suggests that PXR activation should be considered in patients on long-term PXR agonistic antiretroviral drugs (21). Similarly, Cobicistat (COBI) is the backbone of multiple regimens for antiretroviral therapy in AIDS patients. PXR (and CAR) modulate COBI hepatotoxicity through the CYP3A4-dependent pathways (89). The widely used anti-inflammatory drug hypocretin has also been reported as an agonist of PXR, the activation of PXR is followed by upregulation of the downstream proteins CYP3A11, CYP2B10, and organic anion transporter 2 (OATP2), which can also stimulate nuclear migration of YAP, leading to lipid accumulation (10). In addition to the accumulation of triglycerides in the liver of transgenic mice, PXR activation down-regulates hepatic PPARα activity and fibroblast growth factor 12 (FGF21) circulation, which could participate in the pleiotropic role of PXR in energy homeostasis (90).

Carnitine palmitoyltransferase 1A (CPT1A) and mitochondrial 3-hydroxy-3-methylglutaryl-coenzyme A synthase 2 (HMGCS2) are two important enzymes which involved in β-oxidation and ketogenesis. In the absence of insulin, the winged helix/forkhead transcription factor FoxA2 activates transcription of CPT1A and HMGCS2 (91). Insulin induces phosphorylation and exonucleation of FoxA2, which activates FoxA2 and suppresses transcription of CPT1A and HMGCS2 (92). Nakamura et al. showed that PCN down-regulates transcription of CPT1A and HMGCS2 in wild-type mice, but not in Pxr knockout mice. The mechanism may be that PXR directly binds to FoxA2, thus inhibits the activation of CPT1A and HMGCS2 genes (71). **Figure 5B** illustrates the overall mechanism of PXR in cholesterol metabolism.

Cholesterol is essential for the formation of cell membranes, bile acids and steroid hormones. Oxidized cholesterol metabolites are cytotoxic and are a risk factor for atherosclerosis. Cholesterol detoxification protects the body from producing excess cholesterol. In most tissues, the mitochondrial sterol 27-hydroxylase (CYP27A1) is an essential molecule for cholesterol shearing and hydroxylation. Li et al. found that PXR activated CYP27A1 and the cholesterol efflux transport proteins, ATP binding cassette (ABC) subfamily A member 1 (ABCA1) and subfamily G member 1 (ABCG1) in enterocytes (93).

Fibroblast growth factor (FGF) 15 plays a crucial role in the regulation of metabolism. Some findings suggest that PXR may negatively regulate FGF15 expression. In high fat diet (HFD)-fed Pxr knockout mice, intestinal FGF15 expression levels were significantly elevated and total lipids in feces were significantly increased compared with HFD-fed wild-type mice. These represent PXR as a potential therapeutic target for the treatment for metabolic disorders such as obesity (94).

Meng et al. experimented with quetiapine as a PXR agonist, a drug commonly used to treat psychiatric disorders. PXR activation stimulated the intestinal expression of cholesterol transporter Niemann-Pick C1-Like 1 (NPC1L1) and microsomal triglyceride transfer protein (MTP), leading to increased intestinal lipid absorption. Thus, NPC1L1 is a known PXR target gene, they identified a DR-1-type PXR response element in the MTP promoter and established MTP as a potentially novel transcriptional target of PXR (95).

High density lipoprotein (HDL) and its major component apolipoprotein A-I (ApoA-I) are involved in cholesterol reversal and associated with a reduced risk of atherosclerosis. ApoA-I and HDL cholesterol levels can be elevated by Pxr agonists in wild-type mice, but not in Pxr knockout mice. Bile acids mediated the downregulation of HDL cholesterol and lipid ApoA-I was completely absent in human Pxr transgenic mice (96). It has also been suggested that PXR has a pro-atherogenic effect. The expression of ABCA1 is reduced in hepatocytes after PXR activation (97). Clinical use of PXR-activating drugs can lead to hyperlipidemia and drug-induced hypercholesterolemia in some patients (98). Future studies will need to further elucidate the pathological role of PXR in hyperlipidemia.

## PXR in bile acid circulation

Synthesized in the liver, bile acid is the end product of cholesterol catabolism and involved in the body’s removal of cholesterol (99). When bile acid excreted by the intestine, it promotes the absorption of cholesterol and fat-soluble vitamins. However, the excess of bile acid is cytotoxic and can lead to pathological cholestasis. Therefore, the level of bile acid needs to be strictly regulated to avoid toxic damage to the body. PXR plays a crucial role in the detoxification of bile acids. PCN reduced lithocholic acid (LCA)-induced toxicity in wild-type mice, but not in Pxr knockout mice, and Pxr transgenic mice were also tolerant to LCA toxicity. The protective effect of PXR can be explained by the regulation of genes involved in bile acid metabolism. The phase II metabolic enzyme SULT2A is a target gene of PXR and is involved in the detoxification of bile acid (100). In addition to regulating bile acid synthesis and metabolism, PXR also regulates the expression of bile acid transfer proteins, such as MRP2 and OATP2 (101, 102).

Drug-induced hepatotoxicity or acute liver failure remains a key issue in clinical medicine. PARP1-dependent poly(ADP-ribosyl)ation plays a key role in cellular stress responses and functions in multiple physiological and pathological processes. Wang et al. used a mouse model of Acetaminophen (APAP)-induced liver failure to investigate whether PARP1-dependent poly(ADP-ribosyl)ation was involved in the metabolic process. The result indicates that PARP1-dependent poly(ADP-ribosyl)ation of PXR in ligand-binding domain activates PXR competitively and solidly, facilitates its recruitment to target gene CYP3A11 promoter, and promotes CYP3A11 gene transcription, thus up-regulating APAP pro-toxic metabolism. Thus, the inhibition of PARP1-dependent poly(ADP-ribosyl)ation might represent a novel approach for the treatment of drug-induced hepatotoxicity (103). Zeng et al. ‘s experiment on palmitate (PA) treatment of HepG2 cells showed a significant reduction in mRNA levels of CYP3A, but the same results were observed in PXR knockout HepG2 cell lines. The above studies suggest that the transcriptional repression of CYP3A is not regulated by PXR. Although the results of the two experiments are controversial, they suggest that PXR interacts with CYP3A in some way (104).

**Figure 6:**
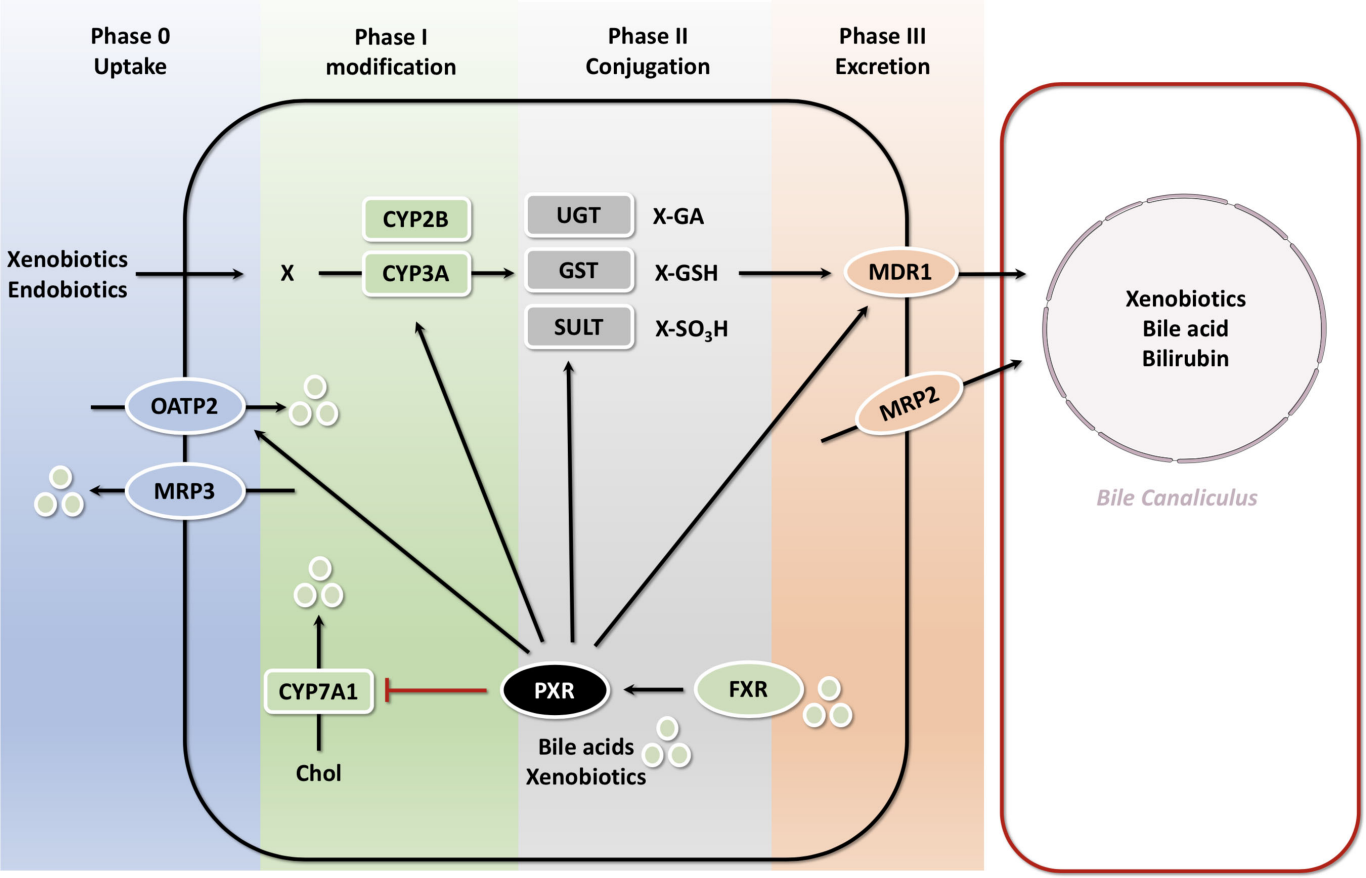
PXR-mediated bile acid transport and metabolism in the hepatocyte with FXR PXR is a dependent transcriptional target of bile acid-activated FXR. PXR can mitigate the harmful effects of toxic bile acids (BA) such as LCA by activation of hepatic detoxification pathways. Activation of PXR induces the uptake of xenobiotics and endobiotics (phase 0), their modification by members of the cytochrome P450 subfamily (phase I), conjugation by GSTs, UGTs and SULTs (phase II) and elimination (phase III) by MRP2, and the multidrug transporter MDR1 which excretion of a wide variety of xenobiotics and endobiotics. PXR can be directly activated by certain bile acids or indirectly *via* transcriptional regulation by FXR. Negative feedback on bile acid metabolism is mediated by inhibition of CYP7A1.

Bilirubin is a degradation product of hemoglobin protein. UDP-glucuronosyltransferase (UGT) binds bilirubin and converts the neurotoxic unconjugated bilirubin into the non-toxic glucuronide bilirubin. Activation of PXR in mice suppresses hyperbilirubinemia. Oleanolic acid (OA) and ursolic acid (UA) activate the transcription of UGT1A1 and some important genes involved in bilirubin detoxification, such as OATP2 and MRP2 through PXR (60, 101, 105). OATP2 mediates the uptake of bilirubin from the blood into the liver and MRP2 facilitates the excretion of conjugated bilirubin into the bile ducts. Although PXR was initially characterized as a xenosensor, the discovery that certain bile acids such as LCA can serve as ligands for both human and mouse PXR provided a link between PXR and bile acid regulation (106). Below we will illustrate the role of PXR in the detoxification of bile acids and the implications in cholestatic disorders. It has been reported that PXR has some interactions with FXR in bile acid regulation. However neither conjugated LCA, nor any of the other conjugated bile acids activate PXR. In addition to direct activation by bile acids, PXR is a dependent transcriptional target of bile acid-activated FXR (107). PXR can mitigate the harmful effects of toxic bile acids (BA) such as LCA by activation of hepatic detoxification pathways. Activation of PXR induces the uptake of xenobiotics and endobiotics (phase 0), their modification by members of the cytochrome P450 subfamily (phase I), conjugation by glutathione S-transferases (GSTs), UDP-glucuronosyl-transferases (UGTs) and sulfotransferases (SULTs) (phase II) and elimination (phase III) by MRP2 (excretion of bilirubin and some bile acids), and the multidrug transporter MDR1 which excretion of a wide variety of xenobiotics and endobiotics. PXR can be directly activated by certain bile acids or indirectly *via* transcriptional regulation by FXR. Negative feedback on bile acid metabolism is mediated by inhibition of CYP7A1. During cholestasis bile acids can also be excreted back into the circulation *via* the sinusoidal ABC-transporters MRP3 and MRP4 (108) (**Figure 6**). Thus, PXR ligands may be potential agents for the treatment of hyperbilirubinemia.

## PXR in vitamin metabolism and bone metabolism

Vitamin K2 is essential for bone formation and is commonly used clinically in the treatment of osteoporosis. Vitamin K2 has been reported to activate PXR and promote the expression of PXR target genes. Treatment of osteosarcoma cells with vitamin K2 increased the expression of the osteoblast markers: bone alkaline phosphatase, osteoprotegerin, bone bridging protein and scaffold Gla protein (109). Vitamin K2 induced the expression of bone markers in primary osteoblasts of wild-type mice, but not in Pxr knockout mice. Ichikawa et al. found that certain PXR target genes are associated with bone function in osteoblasts (110). Besides, the results of Igarashi et al. showed that activation of PXR by vitamin K2 induced the expression of the osteoclastogenic transcription factor muscle segment homeobox 2 (MSX2), which is involved in osteoblast differentiation (111).

In addition, vitamin K2 prevents arterial calcification and atherosclerosis, and adequate intake of vitamin K2 reduces the risk of vascular damage. It is worth mentioning that calcification induced by menaquinone-4 (MK-4), the most common form of vitamin K2 present in animals, can be inhibited by inhibitors of PXR. It was also shown that MK-4 plays an accelerating role in the process of calcification in human aortic valve interstitial cells (HAVICs) through the PXR-BMP2-ALP pathway (112). MK-4 administration also altered mRNA levels of genes involved in drug metabolism (Abca3, Cyp2s1, Sult1b1), and mRNA levels of CYP7A1 and CYP8B1 are similarly changed in human hepatocarcinoma HepG2 cells (113). Besides, MK-4 along with other vitamin Ks, including vitamin K1, has the potential to induce MDR1 and CYP3A4 gene expression. But Pxr knockdown reversed MK-4-mediated stimulation of these genes, indicating the involvement of PXR in this effect. These results elucidate the importance of drug-nutrient interaction mediated *via* PXR (114).

Calcium is an important component of bone development and maintenance. Vitamin D regulates calcium absorption and excretion, and its activated metabolite 1,25(OH)2D3 binds to the vitamin D receptor (VDR). VDR activates 24-hydroxylation mediated by 25-hydroxyvitamin D (3)-24-hydroxylase (CYP24), which is essential in 1,25(OH)2D3 metabolism. Pascussi et al. reported that PXR activation upregulated CYP24 gene expression (115). However, Zhou et al. found that PXR activation inhibited CYP24 gene expression (116). Although the results of the two research are controversial, they suggest that PXR plays a potential function in bone homeostasis and further studies are needed to confirm. Ligand activation of PXR also inhibits the transcription of vitamin D3 25-hydroxylase (CYP2D25) which is an important hydroxylase in 1,25(OH)2D3 biosynthesis (117). Centuries ago, it was found that prolonged treatment with antitussive agents could lead to vitamin D deficiency or chondromalacia in patients. As many antitussive agents are PXR ligands, these results are significant for the prevention of drug-induced chondromalacia in patients.

The treatment and prevention of osteoclast-associated diseases also play a particularly crucial role in addressing problems related to bone metabolism. Guo et al. used common histamine H1 receptor antagonists to experiment *in vivo* and *in vitro*, meclizine reduced osteoclast formation and bone resorption in a dose-dependent manner, while knockdown of PXR with siRNA partially abrogated the osteoclastogenesis inhibition of meclizine (118). Besides, PXR also represses osteoblast differentiation through repression of the Hedgehog signaling pathway, it can repress the Hedgehog signaling-induced genes such as Gli1 and Hhip, and conversely induced the Hedgehog signaling-repressed genes such as Cdon, Boc, and Gas1 (119).

Vitamin E is usually taken as an antioxidant in the daily diet. Vitamin E is metabolized by CYPs-mediated oxidative reactions and then excreted through β-oxidation and binding reactions including glucosylation and sulphation (120, 121). These processes are catalyzed and involved by enzymes and transfer proteins encoded by PXR target genes. Vitamin E activates PXR and may therefore regulate exogenous deleterious genes involved in its own metabolism. A study by Landes et al. using reporter gene analysis showed that PXR can be activated by some forms of vitamin E (122). Vitamin E metabolites were significantly decreased in the urine of wild-type mice following PCN treatment, but not in Pxr knockout mice, suggesting that this was caused by a PXR-mediated decrease in hepatic sterol carrier protein 2 expression and diminished β-oxidation (123). These results have led to widespread interest in investigating potential drug-drug interactions between vitamin E and PXR regulators.

## PXR in endocrine homeostasis

The androgen receptor signaling pathway has an important role in the initiation and progression of prostate cancer. Therefore, androgen blockade is the most effective endocrine therapy for hormone-dependent prostate cancer. The two important PXR target genes, cytochrome P450 (CYP) 3As and hydroxysteroid sulfotransferase (SULT)2A1, function in androgen metabolism. CYP3As is an important enzyme that catalyzes the hydroxylation of testosterone and luteinizing hormone, producing the effects of hormone inactivation. Dehydroepiandrosterone-sulfotransferase 2Al (SULT2A1) is the main SULT isoform involved in androgen sulphonation (124). Zhang et al. reported a PXR-mediated metabolic blockade of androgens. This study revealed that PXR activation decreased androgenic activity and inhibited androgen-dependent prostate regeneration in castrated male rats which received daily testosterone injections to induce CYP3As and SULT2A1 expression (125).

In human prostate cancer cells (LAPC-4 and LA99 cells), treatment with rifampicin (RIF), the human PXR agonist, can inhibit the androgen-dependent proliferation of LAPC-4 cells but had essentially no effect on the growth of non-androgen-dependent homozygous LA99 cells. Downregulation of PXR or SULT2A1 by shRNA or siRNA in LAPC-4 cells abolished the effect of RIF, suggesting that the androgen inhibitory effect of RIF is PXR and SULT2A1 dependent. Thus, PXR may serve as a novel therapeutic target to reduce androgens for the treatment and inhibition of hormone-dependent prostate cancer (125).

Zhai *et al.* showed that PXR plays a crucial role in adrenal steroid homeostasis. The activation of PXR is accompanied by increased cytoplasmic levels of corticosterone and aldosterone and activation of adrenal steroidogenic enzymes such as CYP11a1, CYP11b1, CYP11b2 and 3β-hydroxysteroid dehydrogenase (3β-HSD) (126).

However, adrenocorticotropic hormone of pituitary secretion was normal in Pxr transgenic mice and cortisol was strongly inhibitory to dexamethasone, indicating that normal hypothalamic-pituitary-adrenal axis function even though adrenal steroid homeostasis was severely impaired. Consistent with these observations, some clinical studies have reported that RIF increases urinary steroid secretion and may also lead to misdiagnosis of Cushing’s syndrome (126). Thus, PXR is likely to affect endocrine homeostasis and to function in drug-hormone interactions.

Recently, some studies have linked endocrine disruption, chemical exposure, and cardiovascular disease risk in human, but the underlying mechanisms for this linkage are not clear. Many endocrine disorders involved the activation of the nuclear receptor PXR, and the PXR agonist tributyl citrate induces PXR target gene expression and activates PXR in the small intestine but has no effect on PXR activity in the liver. The mice exposure of tributyl citrate increased plasma total cholesterol and atherogenic LDL cholesterol levels in mice, but not in Pxr knockout mice (23).

## Contribution of chemicals and drugs activating PXR in specific diseases

Recent studies have found that the detoxification system of PXR is a double-edged sword. Although detoxification is a beneficial protective mechanism against toxic compounds, it affects the absorption, distribution, metabolism and elimination of drugs in the body while making the half-life and tissue distribution of drugs in the body unpredictable. At the same time, it may lead to adverse drug reactions during clinical administration, such as reduced drug efficacy, induction of drug toxicity or drug resistance, thus affecting the clinical efficacy and safety of many drugs.

As for the discovery of PXR in aristolochic acid-induced kidney injury and other nephropathy. Atrazine is an herbicide, and environmental exposure to atrazine and its degradation products can cause nephrotoxicity. Atrazine exposure activates the PXR in mice, disrupting CYP450 homeostasis and exacerbating nephrotoxicity. Lycopene supplementation significantly prevented atrazine-induced nephrotoxicity and improved renal injury by modulating CYP450 homeostasis and PXR response (127). In addition, Ochratoxin A is present in food and decreases the survival of human proximal tubular cells and increases the expression of kidney injury molecule 1 (KIM-1).

Ochratoxin A may induce upregulation of PXR gene transcription and cause proximal tubular injury through PXR-related signaling pathways (128). Similarly, Ochratoxin A is also widely present in food and the environment and can cause chronic interstitial nephropathy. Studies have shown that Ochratoxin A does not activate PXR, but when combined with rifampicin, Ochratoxin A can down-regulate PXR gene expression, showing PXR antagonistic effects. In other words, Ochratoxin A is not due to the antagonism itself but due to the downregulation of PXR gene expression (129). In references 129 and 130, Ochratoxin A appear to have contradictory roles in relation to PXR. We suggest that PXR is involved in the regulation of renal drug metabolism and multiple other pathophysiological processes (not limited to the mechanisms explained in the two studies above). The regulation of drug metabolism by PXR *in vivo* is a double-edged sword, both in terms of accelerating toxicant metabolism and thus reducing nephrotoxicity, and in terms of accelerating drug metabolism and mediating drug-drug interactions. PXR is expected to be a therapeutic target in the pathogenesis of various kidney diseases, and to drive the process of clinical drug optimization and new drug development. PXR has a protective effect against acute toxicity induced by a high cholesterol diet. In PXR KO mice, high doses of cholestatic cholesterol feed lead to cholestasis and death due to severe liver and kidney failure. PXR signaling pathway protects the body from toxic dietary cholesterol metabolites, and activation of PXR improves acute renal failure associated with cholestatic liver disease (130).

Apart from renal disease, non-alcoholic fatty liver disease (NAFLD) which has a significant gender difference in the incidence during the whole population. In the process of NAFLD disease development, the expression of PXR and its target gene Cyp3a11 is progressively increased (131). Bile salts in human body may increase NAFLD risk by activating PXR receptor (132). However, polychlorinated biphenyls (PCBS), which can activate PXR, exist in the external environment. Wahlang et al. further found in animal experimental studies that Aroclor1260, a mixture of PCBS, aggravated NAFLD in diet-induced obese mice. Exposure to PCBS promotes the transition from hepatic steatosis to steatohepatitis, in part due to PXR activation. In vascular metabolic disease, Bisphenol A which is a basic chemical substance, is widely found in plastics and exposure to it is ubiquitous (133). In population-based studies, higher BPA exposure has been associated with an increased risk of atherosclerosis (88). In a similar way that BPA may increase the risk of atherosclerosis, some drugs in the clinic may increase the risk of cardiovascular disease by increasing circulating atherogenic lipids after PXR excitation. Karpale *et al.* conducted a serum metabolomic analysis in healthy volunteers, and found that administration of the PXR agonist rifampicin increased serum fractions of very low density lipoproteins and low density lipoproteins compared with placebo (134).

According to the latest studies, activation of PXR, the major regulator of drug metabolism and molecular mediator of clinically significant drug-drug interactions, has been shown to induce hypercholesterolemia. PXR may in part mediate hypercholesterolemic effects of drug treatment. In **Table 2**, we summarized the common drugs (as PXR ligand agonists) and their effects on lipid metabolism from five aspects, drug class, drug, mechanism and the influence on cholesterol and PXR.

**Table 2:** Drugs which can increase the cholesterol and their potential to activate PXR.

Drug class	Drug	Mechanism	The influence on cholesterol	The influence on PXR	Reference
**Antibiotic**	Rifampicin	Bacterial RNA synthesis inhibition	CHOL&LDL↑	PXR agonist	(135, 136)
**Anticonvulsant**	Carbamazepine	Blocking of central Na+ channel	CHOL&LDL↑	PXR agonist	(18, 137)
**Antihypertensive**	Lacidipine	Ca2+ channel blocker	LDL↑	PXR agonist	(138)
**Antineoplastic**	Apalutamide	Antiandrogen	CHOL&LDL↑	Possible PXR agonist	(139)
	Mitotane	Adrenal cortex inhibition	CHOL&LDL↑	PXR agonist	(140–142)
	Ruxolitinib	JAK inhibition	CHOL↑	Possible PXR agonist	(98)
	Brigatinib	Tyrosine kinase inhibition	CHOL&LDL↑	Possible PXR agonist	(98)
	Dasatinib	Tyrosine kinase inhibition	CHOL&LDL↑	PXR agonist	(138)
	Nilotinib	Tyrosine kinase inhibition	CHOL&LDL↑	PXR agonist	(143)
**Antipsychotic, atypical**	Quetiapine	Inhibition of D2 and 5-HT2A receptors	CHOL&LDL↑	PXR agonist	(95)
**Antiretroviral**	Efavirenz	Non-nucleoside reverse transcriptase inhibition	CHOL&LDL↑	PXR agonist	(21, 144, 145)
	Etravirine	Non-nucleoside reverse transcriptase inhibition	CHOL&LDL↑	PXR agonist	(146–149)
	Rilpivirine	Non-nucleoside reverse transcriptase inhibition	CHOL&LDL↑	PXR agonist	(146)
	Darunavir	Protease inhibition	CHOL↑	PXR agonist	(21)
	Fosamprenavir	Protease inhibition	CHOL↑	PXR agonist	(150)
	Lopinavir	Protease inhibition	CHOL↑	PXR agonist	(21)
	Ritonavir	Protease inhibition	CHOL↑	PXR agonist	(151)
	Saquinavir	Protease inhibition	CHOL&LDL↑	PXR agonist	(151)
**Barbiturate**	Phenobarbital	GABA stimulation	LDL↑	PXR agonist	(152, 153)
**Immunosuppressant**	Cyclosporin	Calcineurin inhibition	CHOL&LDL↑	PXR agonist	(154)
	Tacrolimus	Calcineurin inhibition	CHOL↑	PXR agonist	(155)
	Dexamethasone	Glucocorticoid receptor activation	CHOL↑	PXR agonist	(10, 156, 157)
**Proton pump inhibitor**	Lansoprazole	Stomach acid reduction	CHOL↑	PXR agonist	(158)
**Stimulant**	Modafinil	Dopaminergic modulation	CHOL↑	PXR agonist	(159)

## Conclusion and perspective

**Figure 7:**
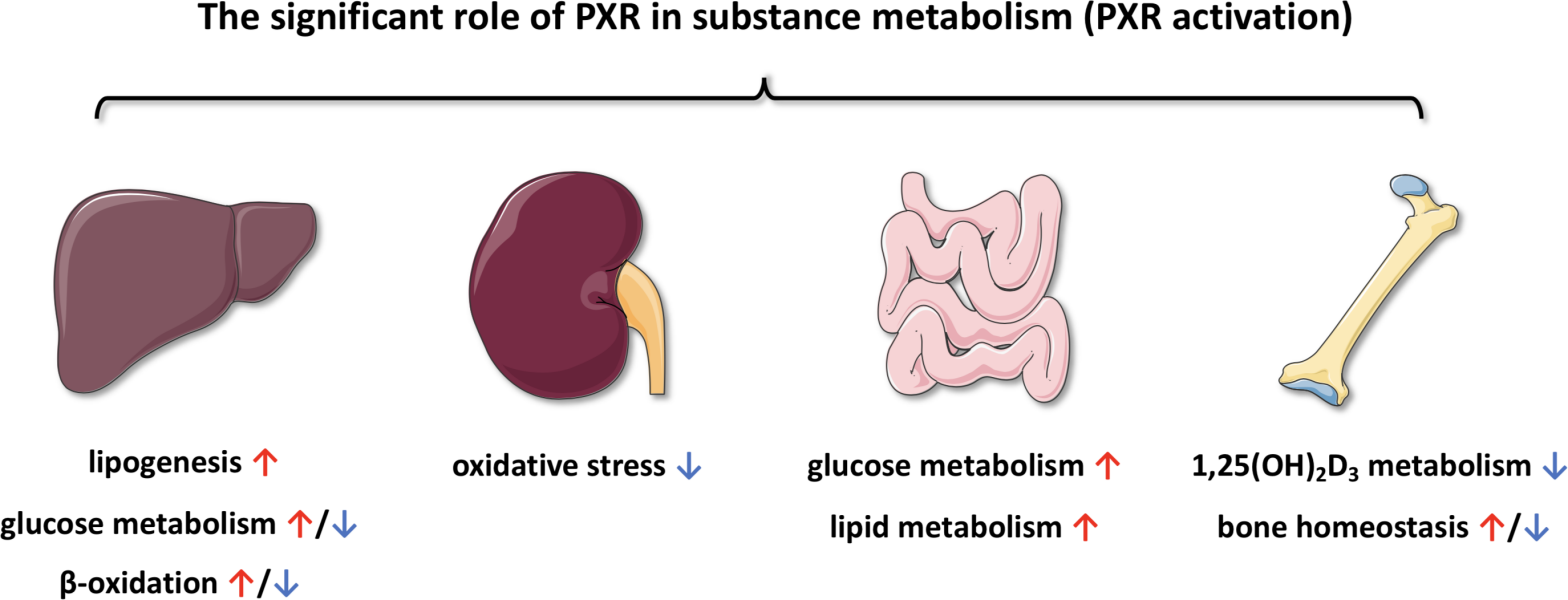
Schematic summary of the functions of PXR in substance metabolism among various organ.

According to accumulated evidence, PXR plays a significant role in substance metabolism (**Figure 7**), including but not limited to glucose metabolism, lipid metabolism and bile acid circulation. At the same time, some of the agonists that have been identified can also be involved in activating PXR during these processes, resulting in different effects. Not only PXR, but also other metabolic NRs may be also involved in the physiological and pathophysiological processes of substance metabolism. As the role of PXR in the regulation of substance metabolism becomes better understood, the use of PXR in the prevention and treatment of human diseases will gradually develop. Another challenge is that although the physiological functions of PXR have been discovered, the endogenous ligands or agonists remain largely elusive. Besides, targeted therapies for metabolic nuclear receptors will also become a new treatment in the future. Overall, PXR is still an attractive target, but the diversity of PXR biology and several pharmacological aspects of PXR modulation should be of concern for the rational therapeutic strategy and novel drug development.

## Author contributions

YL retrieved literature and prepared the initial version of the manuscript and figures. Y-YL retrieved literature and prepared the figures. H-WR edited the initial version of the manuscript and provided suggestions to improve the manuscript. C-JL and Z-XX retrieved literature. Z-LL conceptualized the manuscript and prepared the final version of the manuscript and figures. All authors contributed to the article and approved the submitted version.

## Funding

This work was supported by Education Department of Liaoning Province, China LZ2020023 (to Z-LL), Dalian Young Star of Science and Technology 2019RQ116 (to Z-LL) and Teaching Reform Project Foundation for Undergraduate Innovative Talent Cultivation of Dalian Medical University 111806010301 (to YL). We are also grateful for the support from Liaoning BaiQianWan Talents Program.

## Conflict of interest

The authors declare that the research was conducted in the absence of any commercial or financial relationships that could be construed as a potential conflict of interest.

## Publisher’s note

All claims expressed in this article are solely those of the authors and do not necessarily represent those of their affiliated organizations, or those of the publisher, the editors and the reviewers. Any product that may be evaluated in this article, or claim that may be made by its manufacturer, is not guaranteed or endorsed by the publisher.

## Glossary

**Table:**

NR	nuclear receptor
PXR	pregnane X receptor
FXR	farnesoid X receptor
AF-1	activation function 1
AF-2	activation function 2
DBD	DNA-binding domain
LBD	ligand-binding domain
P	Phosphorylation
Ub	Ubiquitination
Hsp90	heat shock protein 90
RXR	retinoid X receptor
CAR	constitutive androstane receptor
CCRP	CAR cytoplasmic retention protein
RXR	retinoid acid X receptor
XREM	xenobiotic response enhancer module
DR	direct repeat
ER	everted repeat
IR	inverted repeat
MDR1	multi-drug resistance gene 1
MRP2	multi-drug resistance protein 2
GST	glutathione S-transferase
UGT	UDP-glucuronosyltransferase
SULT	sulfotransferase
BA	bile acid
DME	drug-metabolizing enzyme
SMPDL	sphingomyelin phosphodiesterase acid-like
OATP	organic anion transporter
PEPCK	phosphoenolpyruvate carboxykinase
G6Pase	glucose-6-phosphatase
HNF4	hepatic nuclear factor 4
FOXO1	forkhead box protein O1
FOXA2	forkhead box protein A2
CREB	cAMP-response element binding protein
PGC-1α	peroxisome proliferator-activated receptor-gamma coactivator-1α
PCN	pregnenolone-16α-carbonitrile
PKA	activates protein kinase A
VP-hPXR	viral protein human PXR
GLUT2	glucose transporter 2
SGK2	serum/glucocorticoid regulated kinase 2
GCK	glucokinase
EGCG	epigallocatechin-3-gallate
TZD	thiazolidinediones
FABP4	fatty acid binding protein 4
VPA	valproic acid
SREBP	sterol regulatory element-binding protein
LXR	liver X receptor
PPARγ	peroxisome proliferator-activated receptor γ
CPT1A	carnitine palmitoyltransferase 1A
SCD1	stearoyl-CoA desaturease 1
HMGCS2	3-hydroxy-3-methylglutaryl-coenzyme A synthase 2
FGF	fibroblast growth factor
HFD	high fat diet
NPC1L1	Niemann-Pick C1-Like 1
MTP	microsomal triglyceride transfer protein
ApoA-I	apolipoprotein A-I
LCA	lithocholic acid
OA	oleanolic acid
UA	ursolic acid
MK-4	menaquinone-4
VDR	vitamin D receptor
CYP24	cytochrome P450 24
CYP	cytochrome P450
KIM	kidney injury molecular
NAFLD	non-alcoholic fatty liver Disease
AS	atherosclerosis
